# From Cellular Stress to Systemic Adaptation: The Circadian Clock and Stress Response at Cellular and Systemic Levels

**DOI:** 10.3390/ijms27177918

**Published:** 2026-09-05

**Authors:** Isabella Ivankovic, Hanuma Naik Ramavath, Ruifeng Ray Cao

**Affiliations:** 1 Department of Neuroscience and Cell Biology, Robert Wood Johnson Medical School, Rutgers University, Piscataway, NJ 08854, USA; 2 Department of Neurology, Robert Wood Johnson Medical School, Rutgers University, Piscataway, NJ 08854, USA

**Keywords:** circadian clock, stress, glucocorticoid, ISR

## Abstract

The stress response is essential for cellular and organismal survival as it acts as a protective and adaptive mechanism to maintain homeostasis. At the organismal level, physical stressors induce responses in mammals that are mediated primarily by the hypothalamic–pituitary–adrenal (HPA) axis, which regulates glucocorticoid secretion. The HPA axis functions as a circadian-regulated, multi-oscillator system, in which the paraventricular nucleus, the pituitary, and the adrenal gland exhibit intrinsic rhythmicity while remaining coordinated by the output from the suprachiasmatic nucleus. Glucocorticoids act both as stress effectors and systemic zeitgebers that synchronize peripheral clocks. At the cellular level, cellular stressors are sensed by four protein kinases of eukaryotic translation initiation factor 2α (eIF2α) and activate the evolutionarily conserved integrated stress response (ISR), which converges on phosphorylation of Serine 51 on eIF2α. ISR signaling is temporally regulated by the circadian clock and controls time-of-day-dependent protein synthesis. In parallel, ISR pathways feed back onto the circadian clock through transcriptional, translational and epigenetic mechanisms, directly influencing core clock gene expression and stability of circadian oscillations. Physiological ISR activity supports circadian robustness and resetting, whereas excessive ISR activation dampens rhythmic gene expression and destabilizes behavioral rhythms. The current review summarizes recent advances in our understanding of the crosstalk mechanisms between the HPA axis, ISR, and the circadian clock to provide new insights into disease mechanisms and inform chronotherapeutic strategies to target dysregulated HPA and ISR activities and restore temporal homeostasis.

## 1. Introduction

Circadian rhythms are approximately 24 h oscillations that are found in a variety of physiological processes, such as the sleep–wake cycle, glucose metabolism, cardiovascular physiology, immune function, and stress responses. Disruption of these rhythms is associated with a variety of common human diseases. The stress response maintains homeostasis and is essential for animal and cellular survival and well-being. At the systemic level, it operates through the activation of the sympathetic nervous system and the hypothalamic–pituitary–adrenal (HPA) axis. At the cellular level, extracellular and intracellular changes generate the integrated stress response (ISR). These cellular stressors are sensed by ISR regulators, which are specialized protein kinases, including GCN2 (general control non-derepressible 2), PERK (protein kinase R-like endoplasmic reticulum kinase), PKR (Protein kinase RNA-activated), and HRI (heme-regulated inhibitor). Their activities converge on eIF2α (eukaryotic translation initiation factor 2α) to reprogram mRNA translation and the cellular proteome.

Recent research has uncovered significant crosstalk mechanisms between the ISR and the circadian clock, many of which are conserved in a variety of organisms. The two fundamental biological systems coordinate how organisms anticipate predictable environmental changes while adapting to unpredictable challenges. Their interaction represents a deeply conserved evolutionary strategy that optimizes survival, tissue homeostasis, and health. In this review, we summarize the current knowledge regarding how organismal and cellular stress responses interact with the circadian timing system and how these interactions contribute to health and disease. Deciphering the conserved crosstalk between stress response pathways and the circadian clock will establish a unifying framework for understanding how cells integrate predictable environmental cycles with acute physiological challenges. This knowledge will help to identify novel therapeutic strategies for metabolic, neurological, psychiatric, and age-related diseases by targeting the temporal regulation of stress signaling.

## 2. Organization of the Mammalian Circadian System

Life on Earth has evolved under a predictable daily light–dark cycle, giving rise to circadian rhythms that exhibit endogenous oscillations with a period of approximately 24 h. These rhythms enable organisms to anticipate environmental changes and align behavioral, physiological, and metabolic processes with environmental changes throughout the day. In multicellular organisms, the circadian system is organized hierarchically. In mammals, the central pacemaker is located in the suprachiasmatic nucleus (SCN). The SCN receives light input from the retina and resets the phase of downstream behavioral and physiological processes via neural innervation and humoral secretion. Whereas individual SCN neurons can generate their own circadian rhythms via gene expression and electrical activity, the full function of the SCN relies on its network. The SCN network adjusts to external signals, resists genetic disruption, and allows for more precise timing. Therefore, the ability of the SCN to act as a master clock is largely dependent on both its cellular oscillators and network properties [1]. Beyond the SCN, nearly every peripheral tissue harbors its own molecular oscillators, which are normally synchronized by the SCN as well as local cues such as nutrient availability, physical activities, and hormones. Endocrine mediators such as glucocorticoids and melatonin play pivotal roles in transmitting circadian signals across organ systems and enabling coordination between central and peripheral clocks. This coordination ensures that physiological rhythms serve internal metabolic demands aligned with the time of the day.

Circadian rhythms are endogenously driven by self-sustained Transcription–Translation Feedback Loops (TTFLs) within cells. In mammals, the transcriptional activators BMAL1 (brain and muscle ARNT-like 1) and CLOCK (circadian locomotor output cycles kaput) form a heterodimeric complex to activate E-box-mediated transcription of *Period* (*Per1*, *2*, *3*) and *Cryptochrome* (*Cry1*, *2*) genes. PER and CRY form protein complexes in the cytosol and translocate to the nucleus upon reaching a level sufficient to suppress their own gene transcription [2]. Additional feedback loops, such as the REV-ERBα pathway, suppress *Bmal1* transcription and contribute to circadian timing and stability. Circadian clock genes are widely expressed in a variety of tissues and organs. For example, the liver clock plays a major role in regulating glucose and lipid metabolism, as well as helping to maintain daily rhythms in liver function. Unlike the SCN, the liver clock is strongly influenced by external cues such as food intake. While the SCN serves as the central pacemaker, food intake can also reset the liver clock independently of the SCN. For example, changing mealtimes can shift the timing of liver rhythms [3], demonstrating how feeding behavior can directly affect circadian timing in peripheral tissues. These signals help reset and synchronize the liver clock to adapt to metabolic needs and environmental changes. To function properly, the cellular clocks must be synchronized with various extracellular and intracellular signals [4]. Global and cellular stress are gated by circadian timing and serve as strong regulators of the peripheral circadian clocks.

## 3. Mammalian Response to Stress and Circadian Integration

Stress is commonly defined as the result of forces external to the body that disrupt the homeostasis of an organism. Stress can either lead to short-term adaptive functions and have beneficial effects (described as “eustress”) or be maladaptive if experienced chronically or in excess, impairing both recovery and function in organisms (described as “distress”). The stress response has been well established as an evolutionarily conserved mechanism for adaptation and survival [5]. Acute stress is caused by a brief exposure to a stimulus or a singular challenge, such as scenarios of restraint, predator cues, etc. The rapid stress response is mediated by the sympathetic–adrenal–medullary (SAM) axis. This entails immediate release of catecholamines responsible for the “fight or flight” effects [6]. This response typically terminates rapidly. Components of the SAM axis exhibit intrinsic circadian variation in humans, with plasma epinephrine in particular demonstrating diurnal rhythms, indicating that baseline sympathetic–adrenomedullary output is in part time-of-day dependent. Specifically, circulating epinephrine and norepinephrine in humans reach their lowest concentrations during the early morning hours (approximately 2–4 a.m.) and increase during the daytime, reflecting greater sympathetic–adrenomedullary activity during the active phase [7]. While circadian regulation of baseline autonomic and endocrine outputs is well established, recent work demonstrates that both stress-induced corticosterone secretion and neurochemical responses within corticolimbic circuits are gated by circadian phase, with region-specific and signal-specific differences in glucocorticoid and endocannabinoid signaling across the prefrontal cortex, hippocampus, and amygdala. In nocturnal rodents, acute stress responses are generally greatest during the light (inactive) phase and attenuated during the dark (active) phase, with individual corticolimbic regions exhibiting distinct circadian patterns of neurochemical activation rather than a uniform response [8]. These findings emphasize that circadian gating of the stress response is region-specific, such that systemic endocrine responses may be accompanied by distinct temporal responses across individual brain regions.

**Figure 1:**
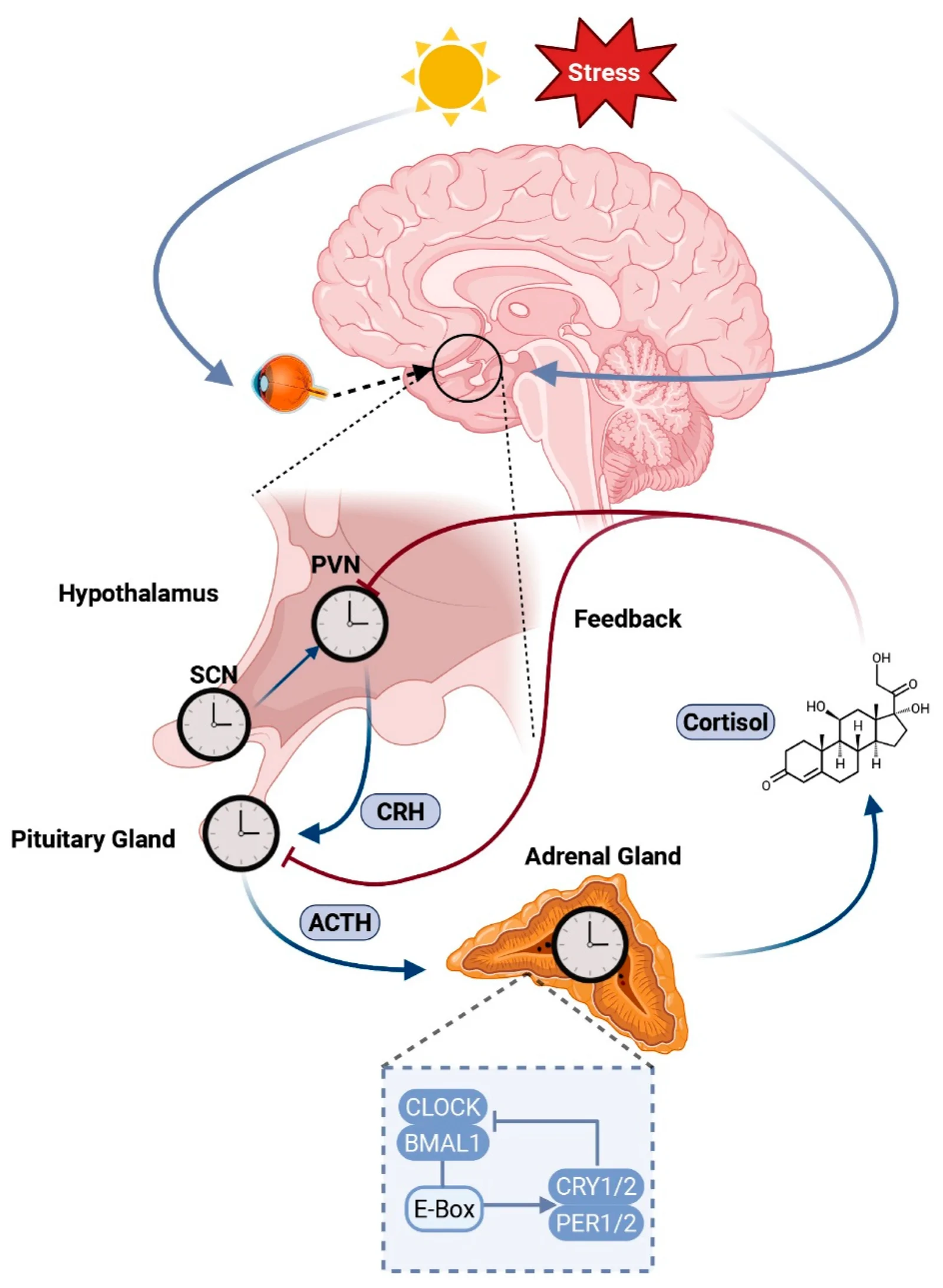
Bidirectional coupling between the circadian clock and the HPA axis shapes time-of-day-dependent stress responsiveness and glucocorticoid signaling. This schematic summarizes the hierarchical organization of circadian and stress systems, emphasizing interactions between the central clock in the suprachiasmatic nucleus (SCN), peripheral clocks in glucocorticoid-sensitive tissues, and the hypothalamic–pituitary–adrenal (HPA) axis. Light input entrains the SCN, which synchronizes peripheral clocks and modulates hypothalamic sensitivity to stress signals. In response to stress exposure, the paraventricular nucleus (PVN) of the hypothalamus releases corticotropin-releasing hormone (CRH) and arginine vasopressin (AVP), driving pituitary adrenocorticotropic hormone (ACTH) secretion and subsequent glucocorticoid release from the adrenal gland. Circulating glucocorticoids, in turn, synchronize peripheral clocks via glucocorticoid receptor (GR) signaling, influencing clock gene expression and phase. This figure highlights how SCN-defined circadian timing gates HPA axis reactivity and shapes daily oscillations in glucocorticoid output, establishing a temporal framework in which stress responses and endocrine signaling are dynamically coordinated across central and peripheral systems. Created in BioRender. Cao, R.R. (2026) https://BioRender.com/kvscwi4.

Chronic stress involves both autonomic activation and responses of the hypothalamic–pituitary–adrenal (HPA) axis. The HPA axis is an essential component of the stress response, rooted in the central nervous system (CNS) and functioning synergistically with autonomic nervous system (ANS) circuitry. Activation of the HPA axis begins in the hypothalamus in response to a stressor; hypothalamic CRH and AVP stimulate ACTH secretion from the anterior pituitary to produce adrenocorticotropic hormone (ACTH). ACTH goes on to stimulate cortisol and corticosterone secretion from the adrenal cortex, which has the effect of both mobilizing glucose and modulating immune and metabolic responses to enable sustained adaptation and optimize chances for survival [9]. The HPA axis is controlled by glucocorticoid negative feedback. In the pituitary gland, glucocorticoid receptors (GRs) reduce ACTH release, while in the hypothalamus, hippocampus, and prefrontal cortex, they further limit stress responses. As a result, stress hormones such as cortisol are released for sustained, chronic, or metabolic stress management [9]. The function of the HPA axis is temporally controlled by the SCN central clock. The activities of the HPA axis exhibit robust circadian oscillations, and systemic cortisol serves as a signal to synchronize peripheral circadian oscillators. Rhythmic secretion of corticosterone is abolished by SCN lesions in rats and basal corticosterone levels become constantly high [10]. Importantly, circadian regulation of the HPA axis functions as an active gating mechanism, such that identical stressors elicit distinct endocrine responses depending on time of day, rather than merely modulating baseline hormonal rhythms [11]. Consequently, stress responsiveness is dynamically adjusted across the circadian cycle, allowing endocrine responses to be tailored to the anticipated physiological demands of the active versus rest phase (Figure 1). Next, we will discuss each component of the HPA axis and their interactions with the circadian clock.

### 3.1. Hypothalamic Integration: Paraventricular Nucleus (PVN)

The paraventricular nucleus (PVN) of the hypothalamus integrates stress, metabolic, and autonomic signals to maintain homeostasis. It controls sympathetic nervous system outflow and hormone release via the PVN–pituitary axis. It also manages energy metabolism, feeding, and stress responses and is a major downstream target of SCN regulation. Early studies have demonstrated that the PVN expresses circadian patterns of electrical activity that are phase-locked to SCN output, and that these rhythms disappear following SCN ablation but can be restored via SCN grafts, indicating that the PVN depends on both neural and humoral (notably AVP) cues for circadian entrainment [12]. Subsequent work has shown that the PVN also participates in shorter-timescale ultradian dynamics, with calcium imaging studies revealing that the subparaventricular zone–paraventricular nucleus (SPZ–PVN) region generates intrinsic ultradian oscillations that propagate to the SCN, likely contributing to rhythmic neuroendocrine secretion, including the pulsatile release of stress hormones [13]. Together, these studies establish the PVN as both a recipient and generator of temporal signals, positioned to translate circadian and ultradian inputs into coordinated hormonal and autonomic outputs.

Cell-type-specific clock gene manipulations reveal that the PVN contains its own local circadian oscillator. Among PVN cell populations, CRH-expressing neurons represent a critical node linking circadian timing to stress response. These neurons exhibit intrinsic rhythms in Per2 expression and calcium activity, and targeted deletion of *Bmal1* in CRH neurons produces marked attenuation of corticosterone rhythmicity [14]. This suggests CRH neurons require an intact molecular clock to help drive coherent HPA-axis output oscillations. Circuit-level analyses from the same study further demonstrate that SCN VIP neurons inhibit PVN CRH neurons to suppress corticosterone release during the daily trough while entraining PVN clock gene rhythms and that appropriately timed CRH neuron activity is required to maintain the amplitude and temporal precision of glucocorticoid release [14]. In parallel, CRH neurons have been implicated in circadian regulation of arousal, with inhibition from SCN GABAergic neurons gating CRH neuron firing across the day, while optogenetic activation of CRH neurons robustly promotes wakefulness through orexinergic pathways [15]. Loss of *Bmal1* within PVN neurons disrupts daily rhythms in activity, feeding, and peripheral gene expression, in part by abolishing the normal diurnal rhythm of oxytocin secretion, indicating that the PVN clock contributes to the propagation of circadian timing signals to systemic physiological outputs [16]. Together, these studies identify the PVN as an integrative circadian output hub, with CRH neurons coupling SCN timing to stress axis activity and arousal, while additional PVN clock-regulated neuronal populations propagate circadian timing signals to broader behavioral and physiological processes.

More recent evidence extends these findings by demonstrating that stress can also act back on the circadian system through PVN-linked circuits. Acute stressors induce phase delays in the SCN through glutamatergic projections from the anterior paraventricular thalamus (aPVT), engaging SCN AVP- and VIP-expressing microcircuits in a manner distinct from classical photic entrainment [17]. The paraventricular thalamus (PVT) serves as a limbic–arousal relay that conveys stress-related signals to the SCN and mediates nonphotic circadian resetting, whereas the hypothalamic paraventricular nucleus (PVN) functions as the principal neuroendocrine and autonomic output hub translating SCN-derived circadian timing into HPA-axis and physiological rhythms. Reciprocal anatomical connections between the PVT and PVN further suggest opportunities for bidirectional communication between stress-processing and endocrine control circuits, although the functional significance of these interactions remains an active area of investigation.

This bidirectional framework provides a mechanistic basis for how chronic stress can destabilize circadian organization and, conversely, how circadian disruption can render animals more vulnerable to maladaptive stress responses. The SCN sets daily windows of HPA reactivity. SCN neurons receive photic input and project via multi-synaptic pathways to both pre-autonomic neurons of the paraventricular nucleus (PVN) and CRH-producing PVN neurons, modulating PVN activity overall in a manner that employs arginine vasopressin (AVP), gamma-aminobutyric acid (GABA), and vasoactive intestinal polypeptide (VIP) neurotransmitters [18]. This results in a time-of-day-dependent CRH release and a subsequent circadian rhythm that manifests in the timely secretion of downstream elements (ACTH and glucocorticoids). SCN output both directly influences CRH neurons and tunes adrenal sensitivity via autonomic pathways, while glucocorticoids released as HPA output feed back on central and peripheral clocks as both stress hormones and zeitgebers (see Section 3.4), thereby functioning as zeitgeber signals within a closed regulatory loop [19].

### 3.2. Pituitary Gland as Circadian Relay

Within the HPA axis, the pituitary functions as the circadian endocrine relay, translating hypothalamic CRH and AVP signals into rhythmic ACTH secretion. Importantly, the pituitary is not merely a passive relay but also contains self-sustaining circadian oscillators. Luciferase reporter experiments reveal robust rhythmicity of *Per1*, *Per2*, and *Bmal1* expression across both anterior and posterior lobes, confirming that most pituitary cells harbor intrinsic clocks that persist ex vivo [20]. Genome-wide profiling demonstrates that hundreds of transcripts within the gland exhibit circadian oscillations, including many involved in hormone synthesis and signaling, suggesting that local clocks contribute to the temporal regulation of endocrine hormone secretion [21].

Comparative analyses further show that the pituitary integrates time cues differently than classical peripheral oscillators. Unlike the liver, which faithfully tracks feeding time, the pituitary displays an intermediate entrainment phenotype, responding to both light-dependent SCN signals and metabolic cues without fully adopting either zeitgeber as dominant [20]. Restricted feeding dampens pituitary clock gene rhythms rather than re-phasing them, and adrenalectomy does not eliminate this partial sensitivity to meal timing, indicating that pituitary entrainment is only weakly dependent on glucocorticoids and instead reflects a more complex integration of neuroendocrine and metabolic inputs [20]. Although SCN-driven CRH signaling provides the dominant circadian input to corticotrophs, intrinsic pituitary clock gene expression is thought to modulate the timing and responsiveness of ACTH secretion, allowing local oscillators to fine-tune endocrine output without overriding hypothalamic control. Together, these findings position the pituitary as a hybrid circadian oscillator: it receives dominant temporal input from upstream SCN-hypothalamic pathways while using its intrinsic clock to fine-tune endocrine responsiveness and hormone secretion. This enables it to help shape the temporal organization of endocrine hormone secretion, including ACTH, TSH, GH, and gonadotropins, thereby coupling central timekeeping to organismal physiology.

### 3.3. Adrenal Gland as Peripheral Oscillator

The adrenal gland functions as both the end organ of the HPA axis and an autonomous peripheral oscillator that directly shapes the timing of glucocorticoid secretion. Glucocorticoid rhythms can persist even when ACTH is held constant, demonstrating that rhythmic adrenal output is not simply a passive reflection of upstream pituitary signaling [22]. More recent molecular studies demonstrate the robust circadian expression of canonical clock components and rhythmic biosynthesis machinery, particularly the steroidogenic acute regulatory protein (StAR), whose oscillatory transcription and translation directly couple the adrenal clock to glucocorticoid production [22,23].

Knockdown of *Bmal1* specifically in the adrenal gland markedly attenuates rhythmic corticosterone production and attenuates behavioral and peripheral gene rhythmicity, indicating that the adrenal clock plays an active role in reinforcing and distributing circadian signals rather than simply responding to central SCN input [24]. This intrinsic rhythmicity operates in parallel with SCN-derived signals, where autonomic innervation via the splanchnic nerve modulates adrenal sensitivity to ACTH, while the central clock provides time-of-day-specific sympathetic drive that shapes the amplitude and phase of steroid secretion [23]. As a result, the adrenal gland integrates central signals via the HPA axis, autonomic, and intrinsic timing cues to produce the robust glucocorticoid rhythm that anticipates the onset of the active period. Collectively, these findings position the adrenal gland as a critical bidirectional node linking circadian timing, stress physiology, and systemic homeostasis. Its intrinsic clock contributes to the generation of circadian glucocorticoid rhythms while modulating how environmental and physiological stressors reshape endocrine timing.

### 3.4. Glucocorticoids as Systemic Zeitgebers

Glucocorticoids (GCs), primarily cortisol in humans and corticosterone in rodents, play a crucial role in coordinating circadian rhythms across the body. GCs typically display a robust circadian pattern, with levels peaking just before the onset of the active phase (morning in humans and evening in nocturnal rodents). As discussed above, this rhythm arises through coordinated regulation by the intrinsic adrenal clock together with circadian input from the HPA axis and sympathetic innervation. Although rhythmic GC secretion can persist even in the absence of ACTH, ACTH pulses are capable of phase-shifting adrenal rhythms, underscoring the dynamic interplay between central and peripheral clocks [25]. Once secreted, GCs act through mineralocorticoid (MR) and glucocorticoid (GR) receptors, with MR providing high-affinity tonic signaling and GR mediating lower-affinity, phasic transcriptional responses [26]. Upon ligand binding, GR translocates to the nucleus, where it regulates thousands of target genes through direct DNA binding or interactions with other transcription factors, which may explain why rhythmic hormone release generates correspondingly rhythmic transcriptional waves across peripheral tissues.

Glucocorticoid oscillations function as potent systemic zeitgebers, in which daily GC surges reset peripheral clocks in organs such as the liver, kidney, lung, and adipose tissue, while the adult SCN clock remains largely insensitive to GC despite its GC receptor expression [27,28]. Adrenal-derived GC signaling in particular is essential for the rhythmic expression of many hepatic genes, and these rhythms are abolished in adrenalectomized animals, while removal of the adrenal alters expression of several core clock genes in the liver, downregulating *Per1* and *Cry1* while upregulating *Rev-Erbα* and *Rorα* [29]. Synthetic glucocorticoids like dexamethasone (DEX) have been shown to strongly induce the expression of core clock genes *Per1*, *Per2*, *Per3*, and *Cry1* and clock-controlled genes such as *Dbp* (D-box binding PAR bZIP transcription factor) in cultured fibroblasts. It has also been shown that DEX suppresses *Rev-Erbα* expression through a mechanism involving the glucocorticoid receptor (GR). Rather than binding directly to the *Rev-Erbα* promoter, GR appears to interact with the CLOCK complex to exert its regulatory effects. In vivo, DEX transiently shifts the phase of circadian gene expression in the liver [27]. Interestingly, the peripheral clock in *Clock* mutant mice entrains to both 24 and 30 h cycles of DEX injections, while wild-type mice only entrain to the 24 h cycle. This suggests that the circadian clock controls systemic responsiveness to glucocorticoid cues [30]. These findings illustrate the complex interplay between systemic hormonal cues and local genetic feedback loops. Through this mechanism, GCs enhance circadian robustness by stabilizing peripheral oscillators [27] against shifts in feeding schedules, environmental perturbations, or jet lag, thereby maintaining internal synchrony [31]. However, the tight coupling between circadian and HPA systems renders the organism vulnerable when stress becomes chronic.

Importantly, glucocorticoid-mediated circadian regulation is tissue and developmental stage-dependent rather than uniform across the body. Although glucocorticoids provide a systemic timing signal, the functional consequences of GR activation differ considerably more among target tissues. Recent evidence demonstrates that the adult SCN expresses functional GRs capable of activating downstream signaling but remains resistant to glucocorticoid-induced clock entrainment, whereas this resistance is absent during fetal development [28]. In the liver, GR signaling interacts extensively with the molecular circadian machinery; however, hepatocyte-specific GR deletion leaves the rhythmicity of most cycling transcripts intact, suggesting that glucocorticoid signaling contributes to, but is not solely responsible for, hepatic circadian gene expression [32]. Together, these findings demonstrate that the circadian effects of glucocorticoids depend on tissue-specific clock architecture and the integration of glucocorticoid signaling with other local and systemic timing cues.

### 3.5. Chronic Stress, the Circadian Clock and Sex Variability

Chronic stress can become maladaptive, entailing persistent sympathetic and HPA activation and leading to secondary effects such as elevated basal glucocorticoids, metabolic strain, immune suppression, and behavioral inflexibility [33]. Dysregulated stress responses often underlie diseases such as anxiety, depression, and metabolic and inflammatory disorders, and animal models demonstrate parallel outcomes across species [34]. Contemporary models further show that HPA hormones act as growth factors for their target glands, producing long-term changes in gland mass and contributing to maladaptive ACTH–cortisol patterns, where impaired GR feedback exacerbates dysregulation [35]. Chronic perturbation of this tightly regulated circadian-gated system carries significant physiological consequences.

Because the stress system and circadian system communicate bidirectionally, disturbances that uncouple these oscillatory networks can propagate system-wide desynchrony, predisposing organisms to metabolic, autoimmune, and mood-related pathologies [36]. Prolonged HPA activation flattens GC rhythms, shifts peripheral phases, and disrupts transcriptional coherence across organ systems. Repeated activation of the HPA axis leads to blunted responsiveness, impaired negative feedback, and altered plasticity, all of which can destabilize downstream clocks and weaken internal synchrony [37]. Aging also compounds this vulnerability. Although the circadian pattern of cortisol release is preserved in elderly individuals, mean cortisol levels increase and adrenal androgen output declines, producing a milieu of heightened glucocorticoid exposure associated with cognitive decline, immunosuppression, and reduced resilience to stress [38,39].

Biological sex represents an additional source of variability in HPA-axis regulation and stress responsiveness. In rodents, females generally exhibit greater ACTH and glucocorticoid responses to acute stress than males, with sex-dependent differences reported at multiple levels of the HPA axis, including PVN CRH and AVP expression, pituitary proopiomelanocortin (POMC) expression, and glucocorticoid-mediated negative feedback. These differences are influenced in part by gonadal hormones, with estrogens and androgens exerting distinct effects on HPA-axis activity and glucocorticoid feedback [40,41]. Importantly, sex differences are also evident in humans but appear more context dependent. Recent evidence demonstrates sex-specific associations between chronic psychological stressors and diurnal cortisol measures, including the cortisol awakening response and diurnal cortisol slope, suggesting that the effects of chronic stress on daily HPA-axis dynamics may depend on both biological sex and stressor type [42]. These findings identify sex as an important biological variable that may modify the interaction between stress exposure and circadian HPA-axis regulation.

Together, the evidence reviewed here demonstrates that stress physiology and circadian timekeeping form an intricately multidirectional system in which the PVN, pituitary, and adrenal gland operate as interconnected circadian-regulated components possessing intrinsic oscillatory properties that both generate and respond to rhythmic signals. Acute stress exploits this architecture to produce adaptive, time-of-day-dependent responses, whereas chronic stress disrupts the coherence of these interconnected clocks, leading to widespread physiological and behavioral dysregulation. The stress axis itself is a multi-oscillator circadian system, and its vulnerability arises from this architecture. Understanding how circadian organization shapes the stress response, and how stress, in turn, perturbs circadian alignment, provides a critical framework for interpreting vulnerability to metabolic, immune, and mood-related disorders across animal systems. However, the relative contributions of individual oscillators to systemic glucocorticoid rhythmicity remain incompletely resolved. Alterations in circulating glucocorticoids may reflect changes in hypothalamic drive, pituitary responsiveness, adrenal sensitivity, or interactions among these regulatory levels. Moreover, as discussed above, these relationships are further modified by biological sex, developmental stage, and tissue-specific sensitivity to glucocorticoid signaling. Distinguishing the relative contributions of these mechanisms will be important for understanding why circadian disruption and chronic stress produce heterogeneous endocrine and physiological outcomes.

## 4. The Integrated Stress Response (ISR) in Cells

**Figure 2:**
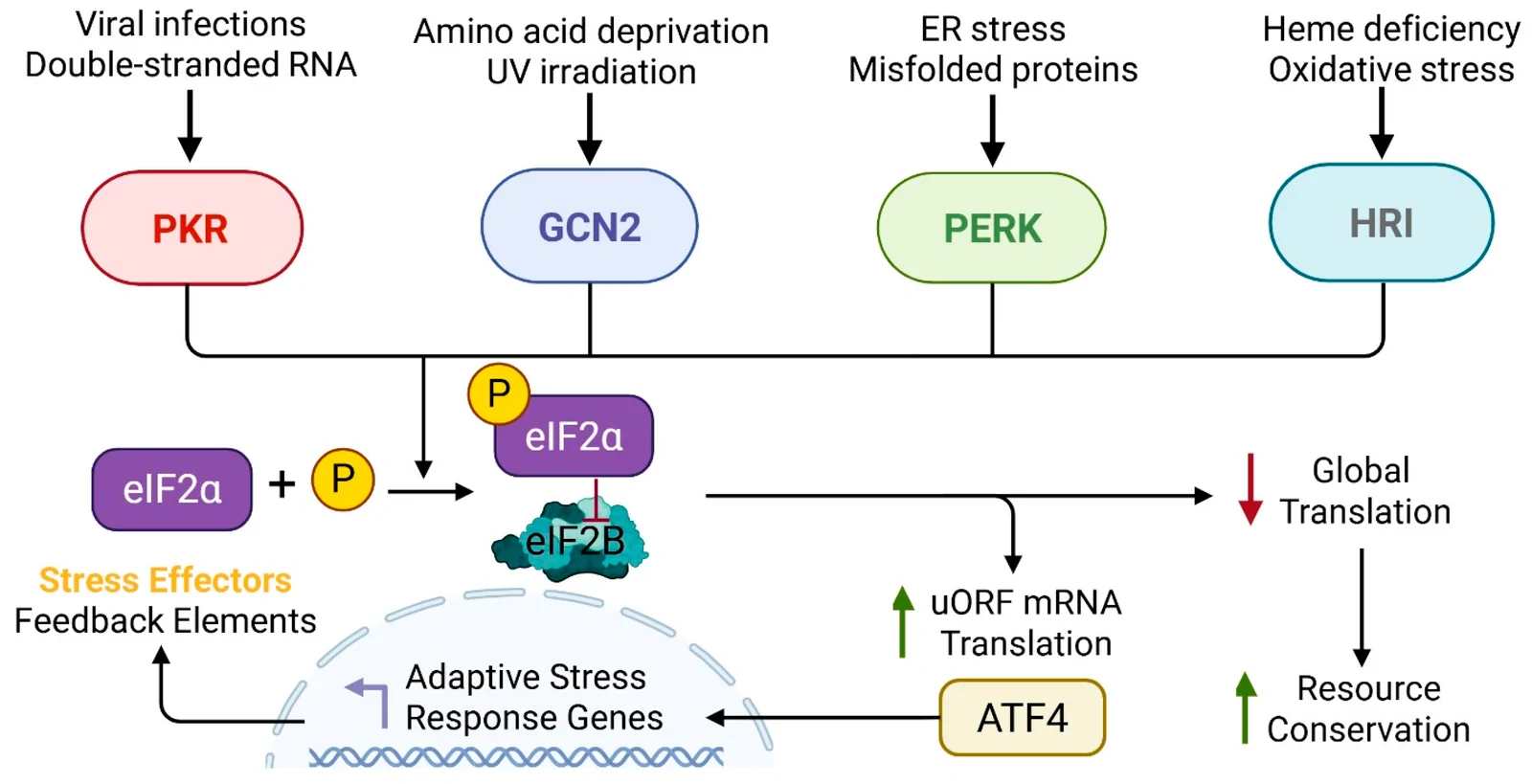
The integrated stress response (ISR) integrates diverse cellular stress signals through eIF2α phosphorylation to regulate translational control. ISR is initiated by four stress-responsive eIF2α kinases, heme-regulated inhibitor (HRI), protein kinase R (PKR), general control nonderepressible 2 (GCN2), and PKR-like endoplasmic reticulum kinase (PERK), each activated by distinct cellular stressors. Despite their distinct upstream inputs, all four kinases converge on phosphorylation of the α-subunit of eukaryotic initiation factor 2 (eIF2α). Phosphorylated eIF2α inhibits the guanine nucleotide exchange factor eIF2B, suppressing global cap-dependent protein synthesis while selectively promoting translation of upstream open reading frame (uORF)-containing transcripts, most notably Atf4. ATF4 subsequently activates transcriptional programs that promote adaptive stress responses, resource conservation, and restoration of cellular homeostasis. Created in BioRender. Cao, R.R. (2026) https://BioRender.com/t6gmwuq.

The integrated stress response (ISR) is a highly conserved cellular signaling pathway that enables cells to adapt to a wide range of internal and external stressors, including nutrient deprivation, mitochondrial dysfunction, oxidative stress, viral infection, aberrant ribonucleic acid (RNA) structures, and protein misfolding within the endoplasmic reticulum (ER). ISR activation is mediated by four stress-responsive kinases, general control nonderepressible 2 (GCN2), protein kinase R-like endoplasmic reticulum kinase (PERK), protein kinase R (PKR), and heme-regulated inhibitor kinase (HRI), which converge on a common target, eukaryotic translation initiation factor 2 alpha (eIF2α) [43]. Phosphorylation of eIF2α suppresses global protein synthesis by inhibiting eukaryotic translation initiation factor 2B (eIF2B), the guanine nucleotide exchange factor required for translation initiation, thereby conserving cellular resources under stress conditions. Concurrently, translation of specific messenger RNAs (mRNAs) containing specialized 5′ untranslated regions (5′ UTRs) with upstream open reading frames (uORFs) is preferentially enhanced. Together, these downstream effectors coordinate adaptive gene expression programs that restore cellular homeostasis (Figure 2).

### 4.1. GCN2

The GCN2 kinase pathway plays a central role in the integrated stress response (ISR), as it senses amino acid deprivation and other similar cellular stress signals. GCN2 is also conserved across eukaryotic cells and serves as a key sensor of amino acid starvation by phosphorylating eIF2α, thereby promoting cell survival under nutrient stress. During amino acid starvation, uncharged tRNAs accumulate and are sensed by GCN2, leading to phosphorylation of eIF2α and activation of the stress response. In yeast, this process requires GCN2 to bind GCN1 and associate it with the ribosome. Recent studies demonstrate that GCN1 directly interacts with the ribosomal protein s10 (Rps10), facilitating transmission of the starvation signal to GCN2. Loss of Rps10 impairs eIF2α phosphorylation and compromises stress adaptation, highlighting the importance of GCN1–Rps10 interaction for effective GCN2 activation during amino acid deprivation [44]. YIH1/IMPACT competes with GCN2 for interaction with GCN1 through the RWD-domain-mediated interaction. YIH1/IMPACT also associate with ribosomes independently of GCN1 and have been proposed to act as regulatory factors by remaining in close proximity to the GCN1–GCN2–ribosome complex. This positioning enables tight control of GCN2 activation, allowing an appropriate stress response during amino acid deprivation [45]. YIH1 primarily inhibits GCN2 by competing with GCN1 binding through RING finger-containing proteins, WD-repeat-containing proteins, and yeast DEAD (DEXD)-like helicases, otherwise abbreviated as the RWD domain. However, YIH1 also binds to actin, and this actin interaction is not required for GCN2 inhibition. As such, actin binding may potentially interfere with the ability of YIH1 to fully block GCN2, leading to possible crosstalk between cytoskeletal components and elements of stress-related translation control [46].

Under UV stress and other conditions such as glucose deprivation and oxidative stress, GCN2 is activated and phosphorylates eIF2α, leading to reduced global protein synthesis and induction of stress-responsive gene expression through its interaction with GCN1. However, IMPACT disrupts GCN1–GCN2 interaction, thereby inhibiting GCN2 activation. Overexpression of IMPACT suppresses GCN2 function, suggesting that IMPACT regulates cellular stress responses by limiting GCN2 activity under diverse stress conditions [47,48]. Activation of GCN2 during amino acid deprivation requires its interaction with the partner protein GCN1 through the RWD binding domain (RWDBD). Disruption of this interaction reduces eIF2α phosphorylation and impairs the stress response. However, specific mutations within the RWDBD can restore or even enhance GCN2 activation. These findings suggest that GCN1-GCN2 interactions regulate eIF2α signaling and stress adaptation [49]. In addition to regulating amino acid stress, the GCN2-eIF2α pathway also helps balance iron levels. In yeast, amino acid supplementation reduces iron uptake through downregulation of iron transporters via a GCN2- and eIF2α-dependent, but GCN4-independent, mechanism. Constitutive activation of GCN2 results in increased iron accumulation and reduced activity of iron–sulfur enzymes, indicating that GCN2 signaling limits iron utilization under amino acid-replete conditions [50]. Together, these findings demonstrate that the GCN2–eIF2α pathway coordinates nutrient and metal homeostasis.

### 4.2. PERK

Endoplasmic reticulum (ER) stress activates three transmembrane sensors, which include activating transcription factor 6 (ATF6), inositol-requiring enzyme 1 (IRE1/ERN1), and PKR-like ER kinase (PERK/EIF2AK3). Together, these sensors initiate the unfolded protein response (UPR), a coordinated signaling network that drives transcriptional and translational reprogramming [51]. The unfolded protein response (UPR) helps cells respond to stress in the endoplasmic reticulum (ER) by activating three sensors, IRE1, PERK, and ATF6, to restore normal ER function. IRE1, via XBP1 (X-box binding protein 1), supports PERK during prolonged stress, promoting cellular adaptation and survival [52]. ER stress activates the PERK pathway, which protects mitochondria by promoting mitochondrial fusion, maintaining their function and metabolism and preventing harmful fragmentation during prolonged stress [53]. PERK protects heart cells from ER stress, and its activation may involve stable oligomer formation rather than stress-induced oligomerization. PEDV (Porcine epidermic diarrhea virus) protein triggers ER stress by activating the PERK and ATF6 pathways but not the IRE1 [54]. The UPR functions to restore ER homeostasis by enhancing protein-folding capacity, promoting degradation of misfolded proteins, and attenuating global protein synthesis. ER stress arises from disruptions in protein folding, triggering activation of these pathways to rebalance cellular proteostasis [55]. Among these sensors, PERK serves as a key mediator linking ER stress to the integrated stress response (ISR). Upon activation, PERK phosphorylates eIF2α, leading to transient suppression of global protein synthesis while selectively enhancing translation of stress-responsive mRNAs such as ATF4. This PERK–eIF2α–ATF4 axis promotes adaptive gene expression programs that support protein folding, redox balance, and metabolic regulation, and disruption of PERK signaling impairs these processes, leading to defects in lipid metabolism, increased oxidative stress, and reduced cell survival [56]. When lacking ATF4, liver cells show significantly more oxidative damage and higher cholesterol synthesis during stress episodes, with blood cholesterol also lowered overall [57]. Although ATF4 is a major downstream effector of PERK, certain stress-induced genes, including CHOP (CCAAT/enhancer-binding protein homologous protein) during ER stress, can also be regulated through ATF6-dependent and complementary IRE1-mediated mechanisms, highlighting the integrated nature of UPR signaling [58].

Beyond its canonical role in ER stress, PERK signaling is also activated in response to diverse cellular perturbations and plays a broader role in regulating translational control during stress. Loss of proper eIF2α phosphorylation disrupts this regulation, leading to sustained inhibition of global protein synthesis. Viral proteins like human cytomegalovirus (HCMV) UL148 (unique long region 148) can trigger the PERK-eIF2α-ATF4 pathway, linking viral infection to UPR and modulation of host cell homeostasis [59]. UL148, together with UL116, regulates the folding and assembly of viral glycoprotein complexes, proper virion maturation, and infection. In this context, PERK activation not only reflects ER stress adaptation but may also facilitate viral protein quality control and assembly [60]. Similarly, toxins such as mycolactone, produced by *Mycobacterium ulcerans*, activate the ISR by blocking secretory mutant 61 (Sec61)-mediated protein translocation into the ER. This induces eIF2α phosphorylation through PERK, GCN2, and PKR, leading to ATF4 activation independently of classical ER stress sensors such as IRE1 or ATF6. While this response is initially protective, prolonged activation shifts signaling toward autophagy and apoptosis via ATF4–CHOP, demonstrating how sustained ISR activation can influence cell fate decisions. Together, these findings highlight PERK as a central integrator of ER-specific and broader cellular stress signals, linking translational control, protein homeostasis, and environmental stress responses. This expanded role positions PERK as a critical node through which diverse stressors, including metabolic imbalance, infection, and toxic insult, may converge, with important implications for how cellular stress pathways interface with systemic processes such as circadian regulation and disease progression.

### 4.3. PKR

PKR (also known as EIF2AK2) is a key kinase within the ISR that phosphorylates eIF2α in response to viral infection and other cellular stressors [61]. Classically, PKR is activated upon binding to double-stranded RNA (dsRNA), enabling it to sense viral infection and inhibit translation of viral mRNAs while promoting apoptosis in severely damaged cells. In addition to viral dsRNA, PKR can also be activated by oxidative stress, inflammatory cytokines, and other environmental stress signals, highlighting its broader role as a cellular stress sensor. Upon activation, PKR phosphorylates eIF2α, leading to a reduction in global protein synthesis while selectively enhancing translation of stress-responsive genes such as ATF4. This, in turn, promotes downstream effectors including ATF3, CHOP, and GADD34, which together regulate cell survival and apoptosis depending on stress severity. Through this mechanism, PKR contributes directly to ISR-mediated translational control and adaptive gene expression. Beyond its role in translation, PKR also modulates additional signaling pathways, including NF-κB and MAPK, thereby linking translational repression to inflammatory and immune responses. Dysregulation of PKR signaling has been implicated in a range of diseases, including neurodegeneration, cancer, and metabolic disorders, underscoring its importance in maintaining cellular homeostasis [62].

Importantly, PKR activation is not limited to viral infection. Although PKR is classically recognized as a sensor of viral double-stranded RNA, it can also be activated by endogenous RNAs, particularly mitochondrial RNAs (mtRNAs). These mtRNAs can form double-stranded structures that activate PKR, leading to eIF2α phosphorylation and modulation of translation and stress-related signaling. Tight regulation of PKR activity is essential, as disruption of PKR phosphatases can lead to uncontrolled activation and apoptosis even in the absence of infection. This indicates that PKR has the capacity to function more broadly as a sensor of mitochondrial and nuclear stress signals [63]. Structurally, PKR is tightly controlled by its two N-terminal dsRNA binding domains (dsRBD1 and dsRBD2), which sense viral or endogenous double-stranded RNA. Activation involves dimerization and autophosphorylation, including phosphorylation of critical residues such as Thr446 and Thr451. The dsRBD1 domain also contributes to maintaining basal kinase activity, and deletion of inhibitory sequences within this region can result in constitutive PKR activation independent of dsRNA. Additionally, alternative activation mechanisms exist, including caspase-mediated cleavage that removes inhibitory domains, further demonstrating that PKR can be regulated through both RNA-dependent and RNA-independent mechanisms [64,65]. Together, these findings establish PKR as a multifaceted regulator of cellular stress responses, integrating signals from viral, mitochondrial, and inflammatory stress pathways to coordinate translational control and cell fate decisions. This broad functionality positions PKR as a critical node linking immune activation and cellular stress signaling, with potential relevance for how stress responses interface with systemic regulatory processes and disease progression.

### 4.4. HRI

HRI (also known as EIF2AK1) was initially identified for its role in sensing heme deficiency. HRI is now recognized as a broader sensor of cytosolic proteotoxic stress, including oxidative stress, proteotoxic and mitochondrial stress signaling. HRI contributes to a cytosolic UPR and additionally participates in mito-nuclear communication, linking protein quality control and cellular defense pathways through eIF2α phosphorylation and ATF4 induction [66]. In erythroid cells, HRI plays a critical role in coordinating translation during iron and heme deficiency. Ribosome profiling reveals that HRI not only promotes ATF4-dependent ISR gene expression but also suppresses translation of cytosolic and mitochondrial ribosomal proteins, leading to a reduction in overall protein synthesis. Loss of HRI under iron deficiency conditions leads to cytoplasmic proteotoxic stress, mitochondrial respiration, and defective erythroid differentiation [67]. However, HRI-driven ISR also activates regulators like GRB10, linking translational control to erythropoiesis. Recent studies reveal that HRI also functions as a negative regulator of PTEN-induced kinase 1 (PINK1) dependent mitophagy, a pathway essential for mitochondrial quality control. Knockdown of HRI or its activator DAP3 binding cell death enhancer (DELE1) enhances mitochondrial damage-induced stabilization of PINK1, leading to increased phosphorylation of ubiquitin and Ras-related in brain 8A (RAB8A), and promotes mitophagy. ISR inhibition using an integrated stress response inhibitor (ISRIB) mimics these effects, suggesting that the DELE1-HRI-ISR axis restrains mitophagy during mitochondrial stress. These reports suggest a novel link between ISR and mitochondrial turnover, which may have therapeutic relevance for Parkinson’s disease [68]. HRI activation during iron deficiency has recently been linked to the mitochondrial protein DELE1, whose stability is regulated by iron-dependent mitochondrial import [66,68].

Under normal conditions, DELE1 is rapidly degraded by the mitochondrial matrix protease LONP1. During iron depletion, mitochondrial import of DELE1 is impaired, allowing it to accumulate on the mitochondrial surface, where it activates HRI and initiates ISR signaling. Loss of the DELE1–HRI pathway increases cell death in iron-deficient erythroid cells, highlighting its protective role in iron-sensitive lineages [69,70]. Consistent with this, HRI has emerged as a key eIF2α kinase linking mitochondrial stress to ATF4 activation. During mitochondrial dysfunction, the metalloprotease OMA1 cleaves DELE1, enabling its accumulation in the cytosol and subsequent activation of HRI. This OMA1–DELE1–HRI axis is essential for ATF4-mediated stress adaptation, and disruption of this pathway triggers alternative responses, including selective induction of molecular chaperones [69,71]. In addition to its role in mitochondrial stress signaling, the HRI–eIF2α–ATF4 pathway also suppresses mechanistic target of rapamycin complex 1 (mTORC1) activity, particularly in erythroid cells. Loss of HRI disrupts this coordination, impairing erythroid differentiation despite partial rescue by pharmacologic mTORC1 inhibition. Persistent oxidative stress and globin aggregation in HRI-deficient models indicate that HRI has nonredundant functions beyond mTORC1 regulation. Notably, dietary iron repletion restores this balance by suppressing both HRI and mTORC1 signaling, underscoring HRI’s role as a key regulator of translational control during erythropoiesis under iron-limited conditions [72,73]. Together, these findings position HRI as a critical integrator of mitochondrial, iron-dependent, and translational stress signals, coordinating adaptive responses that maintain cellular and metabolic homeostasis. This expanded role highlights HRI as an important mediator linking intracellular stress sensing to broader physiological processes, with potential implications for diseases involving mitochondrial dysfunction, anemia, and systemic stress adaptation.

## 5. ISR and the Circadian Clock

The ISR and the circadian system are two highly conserved adaptive systems that enable cells to maintain homeostasis in response to environmental and physiological challenges. While the circadian clock anticipates predictable daily fluctuations in cellular demand, the ISR orchestrates rapid translational reprogramming in response to acute stress. Despite their central roles in regulating protein homeostasis, metabolism, and neuronal function, these systems have traditionally been studied independently, and how these pathways interact remains largely unknown. Emerging evidence indicates that they are mechanistically intertwined, with ISR signaling being regulated by the circadian clock and feeding back to regulate the circadian clock function. Defining the molecular crosstalk between the ISR and the circadian clock has broad implications for understanding the pathogenesis of neurodevelopmental, neurodegenerative, psychiatric, and metabolic disorders, in which both circadian dysfunction and chronic ISR activation are common features. This new knowledge may establish a foundation for developing chronotherapeutic strategies that target stress-response pathways to improve disease outcomes.

### 5.1. Circadian Regulation of ISR

A growing body of work demonstrates that the timing and magnitude of the ISR are regulated by the circadian clock. Central to this relationship is the daily rhythmic phosphorylation of eIF2α, a key event in ISR signaling that controls translation initiation. In the SCN, the level of eIF2α phosphorylation exhibits significant circadian oscillations, high during the day and low at night. The eIF2α kinase GCN2 is required for this oscillation, as the circadian rhythmicity of eIF2α phosphorylation is dampened in the SCN of *Gcn2* mutant mice [74]. In *Neurospora crassa*, levels of eIF2α phosphorylation exhibit similar patterns of circadian oscillation, peaking during the subjective day [75]. The rhythmic eIF2α phosphorylation in *Neurospora* depends on the activity of CPC-3, the fungal homolog of the eIF2α kinase GCN2, indicating that circadian regulation of ISR is evolutionarily conserved. Mechanistically, circadian regulation of eIF2α phosphorylation is closely linked to cellular nutrient sensing, e.g., the balance between charged and uncharged transfer RNAs (tRNAs) [75]. Karki et al. demonstrated that rhythmic accumulation of uncharged tRNAs across the circadian cycle contributes to time-of-day-specific activation of CPC-3, thereby driving oscillations [75]. In addition to the activity of CPC-3, circadian clock regulation of eIF2α activity requires dephosphorylation by PPP-1 phosphatase at night [76]. This, in turn, imposes temporal gating on translation, with elevated p-eIF2α during the subjective day leading to reduced global translation and selective synthesis of specific proteins [77]. Circadian clock control of tRNA synthetases drives rhythms in uncharged tRNAs, leading to rhythmic CPC-3 activation [78].

Notably, disruption of rhythmic eIF2α phosphorylation does not abolish the core *Neurospora* transcriptional oscillator, but instead alters downstream rhythmic protein production, indicating that ISR-mediated translational control functions in part as a circadian output mechanism that refines temporal gene expression in *Neurospora* [75]. Under conditions of amino acid limitation, accumulation of uncharged tRNAs activates CPC-3 through interactions with the adaptor protein GCN1 and the ribosome [79]. These interactions are rhythmic and depend on fluctuations in uncharged tRNA levels, providing a mechanistic link between metabolic state and circadian timing. When uncharged tRNA levels are constitutively elevated, rhythmic CPC-3 activity and its association with ribosomes are lost, demonstrating that temporal regulation of the translational environment is essential for circadian control of ISR signaling [79]. Together, these findings support a model in which the circadian clock orchestrates translational dynamics by modulating ISR components, ensuring that protein synthesis is temporally aligned with cellular energy availability and metabolic demands.

### 5.2. Regulation of Circadian Clock Function by the ISR

In addition to being regulated by the circadian clock, recent work has started to uncover the critical role of the ISR in regulating circadian clock physiology. In the mammalian system, GCN2-dependent phosphorylation of eIF2α promotes preferential translation of *Atf4* mRNA, which contains uORFs that enable its translation [75]. Once translated, ATF4 acts as a transcriptional regulator that directly interfaces with circadian gene networks. Pathak et al. demonstrated that ATF4 binds to regulatory elements within multiple clock genes, including *Per2*, and enhances *Per2* transcription [74]. In the SCN, rhythmic eIF2α phosphorylation leads to time-of-day-dependent translation of ATF4 and subsequent modulation of clock gene expression. The clock protein PER1 and PER2 levels and oscillations in the SCN are both dampened in *Gcn2* and *Atf4* mutant mice. As a result, mice exhibit lengthened circadian period and impaired behavioral rhythmicity. Conversely, increased eIF2α phosphorylation shortens the circadian period in mice and cells, indicating that the level of ISR activity can bidirectionally regulate the length of the circadian period. These findings establish ISR-mediated translational control as a ubiquitous mechanism to regulate the function of the central and peripheral circadian clocks.

Beyond transcriptional regulation through ATF, ISR signaling may also influence circadian function through modulation of histone acetylation, particularly under metabolic stress. Liu et al. examined the role of the ISR signaling pathway during amino acid starvation in *Neurospora*, revealing that ISR activation is required to maintain robust circadian rhythms under nutrient-limited conditions [80]. In this context, activation of CPC-3 and its downstream effector CPC-1 (functional homolog of ATF4) promotes transcription of the core clock gene *Frequency (Frq)* by facilitating a permissive chromatin environment at its promoter. Specifically, CPC-1 recruits the histone acetyltransferase GCN5, leading to increased acetylation of histone H3 and enhanced binding of WCC (White Collar Complex), the primary transcriptional activator of *Frq*. Loss of CPC-3 or CPC-1 disrupts this regulatory axis, resulting in reduced histone acetylation at the *Frq* promoter, impaired transcriptional activation, and breakdown of rhythmic gene expression under amino acid starvation. Importantly, pharmacological inhibition of histone deacetylases partially rescues rhythmicity in these mutants, further supporting the conclusion that ISR signaling maintains circadian transcription through chromatin remodeling mechanisms [80]. These findings extend the role of the ISR beyond translational control, demonstrating that it can directly influence the epigenetic landscape of core clock genes to preserve rhythmicity under metabolic stress. Together, these cross-species studies reveal that ISR is a conserved circadian regulator that plays an integral role in the regulatory network of the circadian clock machinery.

### 5.3. Deregulation of ISR and Circadian Clock Dysfunction

While physiological ISR signaling can support circadian timing and clock robustness, emerging evidence supports that sustained or excessive activation of ISR pathways disrupts clock gene expression and circadian clock function. Under ER stress, activation of the PERK kinase leads to phosphorylation of eIF2α and induction of downstream stress-responsive programs [81]. Bu et al. identified a specific pathway through which the PERK-miR-211 axis suppresses the circadian clock and promotes cancer cell survival [82]. In this model, PERK activation induces the expression of microRNA-211, which in turn targets the core clock transcription factors *Bmal1* and *Clock*. Suppression of these components disrupts the transcriptional–translational feedback loop that underlies circadian oscillation, leading to phase shifts and reduced amplitude of rhythmic gene expression. These effects were observed both in cultured cells and in vivo, where ER stress induces phase shifts in *Bmal1* and *Clock* expression in the liver. Importantly, inhibition of PERK signaling prevents these effects, demonstrating that the observed circadian disruption is mechanistically dependent on ISR overactivation [82]. This mechanism appears to be functionally relevant in pathological contexts such as cancer, where suppression of circadian regulation may repress differentiation and promote cancer cell proliferation.

**Figure 3:**
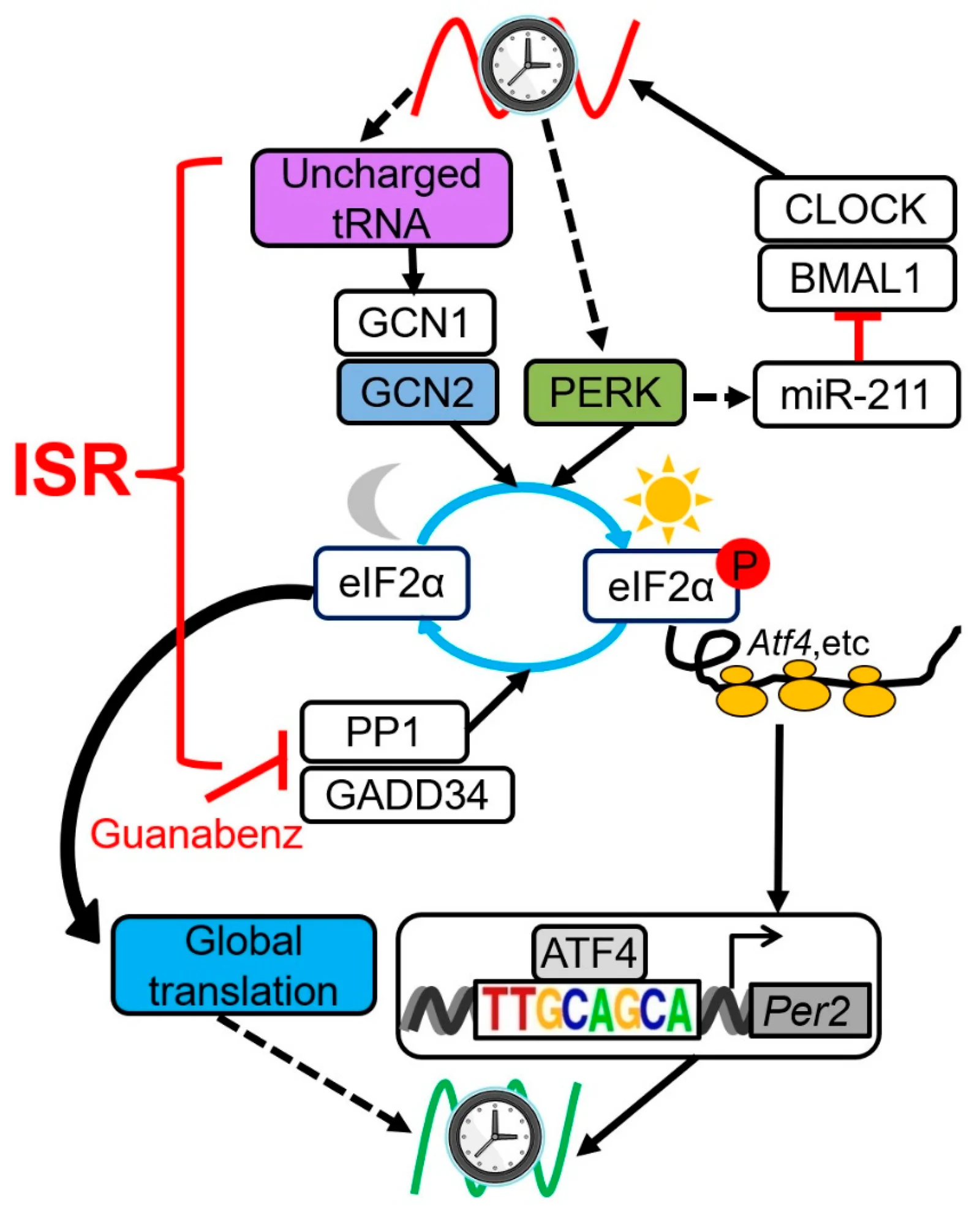
The crosstalk mechanisms between the integrated stress response (ISR) and the mammalian circadian clock. This conceptual model illustrates how nutrient availability modulates circadian rhythmicity through activation of the General Control Nonderepressible 2 (GCN2)-dependent ISR activation. The level of uncharged tRNAs is rhythmically regulated by the circadian clock. Uncharged tRNAs activate GCN2, resulting in phosphorylation of the α-subunit of eukaryotic initiation factor 2 (eIF2α) and preferential translation of specific transcripts including *Atf4*. ATF4 subsequently promotes transcriptions of clock genes including *Per2* via ATF4 binding motifs, which in turn feeds back to the core circadian machinery to regulate speed and robustness of the circadian clock, linking ISR to circadian timing. In the cancer model, PERK activation induces the expression of microRNA-211, which in turn represses the core clock transcription factors BMAL1 and CLOCK and leads to phase shifts and reduced amplitude of rhythmic gene expression. Guanabenz binds directly to GADD34 (Growth Arrest and DNA Damage-inducible protein 34) and inhibits protein phosphatase 1 (PP1), leading to sustained eIF2α phosphorylation and clock disruption. Created in Microsoft 365 enterprise.

Complementing these findings, pharmacological studies further demonstrate that ISR hyperactivation is sufficient to disrupt circadian rhythms in WT mice. Lin et al. investigated the effects of guanabenz, a compound known to enhance ISR signaling by promoting sustained eIF2α phosphorylation, on circadian function in cells and mice [83]. Guanabenz treatment resulted in increased levels of phosphorylated eIF2α in both cultured fibroblasts and the mouse brain, including the SCN. This was accompanied by shortening of the circadian period, reduced amplitude of behavioral rhythms, and decreased locomotor activity during the active (dark) phase. At the molecular level, guanabenz increases *Per1* and *Per2* expression and disrupts circadian oscillations of the clock protein PER1 and PER2 within the SCN, indicating a direct impact on the central pacemaker [83]. These significant findings reinforce the idea that persistent and exacerbated eIF2α phosphorylation perturbs the stability and function of the core clock, in contrast to the rhythmic and tightly regulated ISR signaling observed under physiological conditions. Together, these studies demonstrate that the relationship between ISR and the circadian clock is dependent on context, underscoring the importance of considering both the magnitude and source of ISR activation when evaluating its effects on circadian systems. Whereas rhythmic ISR activity at a physiological level is integral for normal clock regulation and required for clock functions, pathological stress or pharmacologically enhanced ISR signaling can destabilize circadian oscillations and impair circadian clock function (Figure 3).

### 5.4. Potential Crosstalk Between the HPA Axis and ISR in Circadian Regulation

There is little information on the potential interactions between the HPA axis and ISR in regulating the circadian clock. In vitro studies show that glucocorticoids can induce the phosphorylation of eIF2α in specific cell types. For example, cortisone can activate ISR by activating GCN2 and promote stress granule assembly when combined with vinorelbine treatment in U2OS cells and breast cancer organoids [84]. Dexamethasone induces the phosphorylation of eIF2α, resulting in the translational upregulation of ATF4 via activation of HRI, and induces apoptosis in multiple myeloma cells [85]. Although experimental evidence is yet to be established, a potential synergistic role of GCN2 and the HPA axis in regulating the central circadian timing system was studied by computational modeling. The authors concluded that the indirect activation of GCN2 by the HPA axis provided a potential mechanism whereby the SCN senses stress signals from the HPA axis, supporting the complex interactions between ISR sensing and circadian rhythm regulation [86].

## 6. Chronotherapeutic Strategies Targeting the HPA Axis and ISR

Chronotherapeutic strategies targeting the HPA axis and ISR have been applied to improve efficacy and reduce side effects of glucocorticoids. For example, cortisol naturally peaks drastically in the early morning to provide an “alertness drive”. Patients with adrenal insufficiency or congenital adrenal hyperplasia traditionally took standard hydrocortisone tablets upon waking, which failed to mimic the natural pre-waking surge. Modern chronotherapy uses delayed-release or modified-release hydrocortisone taken at bedtime [87]. The drug releases in the middle of the night, successfully replicating the physiological morning cortisol peak and significantly improving metabolic and quality-of-life outcomes [88]. Symptoms of rheumatoid arthritis including joint stiffness and pain peak in the morning due to a nighttime surge of pro-inflammatory cytokines [89]. Chronotherapy with low-dose prednisone (designed to release exactly at 2:00 a.m. while the patient sleeps) targets this HPA-axis/immune interface precisely when cytokine production begins, offering superior symptom relief compared to morning dosing [90,91].

Administering synthetic glucocorticoids at the wrong time of day (e.g., split into multiple doses coinciding with evening meals or bedtime) triggers a massive negative feedback loop. This artificially suppresses the endogenous HPA axis, leading to long-term adrenal atrophy, severe metabolic syndrome, and systemic immune suppression. Because every patient has a distinct internal “chronotype” (e.g., night owls vs. early birds), the precise peak of HPA axis sensitivity varies. Applying a uniform, “one-size-fits-all” chronotherapeutic drug schedule can inadvertently cause circadian misalignment in certain individuals, causing morning anxiety or severe sleep disturbances.

Chronotherapeutic delivery of cancer drugs or small-molecule ISR inhibitors is timed to hit cells during their peak physiological vulnerability window—maximizing cancer cell death while sparing healthy tissues whose translation machinery is at a different phase of the clock [92]. Mistimed eating (like late-night snacking) serves as a potent circadian stressor that triggers ER stress and activates the ISR in the liver [93]. Time-restricted feeding (TRF) is used as a non-pharmacological chronotherapeutic strategy. Restricting food to the natural active phase aligns the central clock with peripheral metabolic tissue clocks, suppressing chronic ISR activation and reversing insulin resistance [94]. When circadian disruption causes the ISR and the HPA axis to remain chronically active at night, it creates a persistent catabolic state. Nighttime cortisol elevations and continuous eIF2α phosphorylation permanently halt muscle protein synthesis while accelerating fat accumulation. Overcoming this specific therapeutic barrier requires complex, multi-tiered interventions (combining light therapy, strict meal windows, and strict sleep hygiene) rather than a single medication [95].

## 7. Conclusions

Circadian rhythms temporally organize metabolism, physiology, and behavior through a hierarchical system, in which the central SCN clock synchronizes peripheral clocks via neural, endocrine, and behavioral signals. The circadian network extends beyond timekeeping, integrating metabolic and stress-responsive pathways to maintain temporal homeostasis. At the organismal level, the HPA axis functions as a circadian-regulated, multi-oscillator system, in which the PVN, pituitary, and adrenal gland contribute intrinsic rhythmicity while remaining coordinated by SCN output. Glucocorticoids play a central role in this architecture, acting both as stress effectors and systemic zeitgebers that synchronize peripheral clocks.At the cellular level, the ISR serves as a conserved mechanism of adaptation, in which eIF2α kinases including GCN2, PERK, PKR, and HRI converge on eIF2α to regulate translational output and promote stress-responsive gene expression. ISR signaling is temporally regulated, with circadian modulation of eIF2α phosphorylation and downstream effectors such as ATF4 enabling time-of-day-dependent control of protein synthesis and metabolic adaptation. In parallel, ISR pathways feed back onto the circadian clock through transcriptional, translational and epigenetic mechanisms, directly influencing core clock gene expression and stability of circadian oscillations. Physiological ISR activity supports circadian robustness and adaptive flexibility, whereas sustained or excessive ISR activation disrupts clock integrity, dampens rhythmic gene expression, and destabilizes behavioral and endocrine rhythms. Understanding the temporal dynamics of stress–clock interactions may provide a framework for chronotherapeutic strategies aimed at restoring physiological timing and improving outcomes for treating a variety of human diseases.

## Data Availability

No new data were created or analyzed in this study. Data sharing is not applicable to this article.
